# Editorial: Enzymes from Extreme Environments

**DOI:** 10.3389/fbioe.2016.00024

**Published:** 2016-03-07

**Authors:** Noha M. Mesbah, Felipe Sarmiento

**Affiliations:** ^1^Faculty of Pharmacy, Suez Canal University, Ismailia, Egypt; ^2^Swissaustral USA LLC, Athens, GA, USA

**Keywords:** extremozymes, enzymes and coenzymes, biotechnology, extremophiles, biocatalysis

**The Editorial on the Research Topic**

**Enzymes from Extreme Environments**

Enzymes are nature’s biocatalysts and are equipped with high catalytic activity and remarkable substrate specificity. Enzymes catalyze the metabolic reactions necessary for all of life’s processes and are able to catalyze reactions at much higher rates than would be possible without their action. The majority of enzymes are optimized to perform under physiological conditions or conditions considered normal for mesophilic, neutrophilic microorganisms (de Carvalho, 2011). However, a particular set of enzymes called “extremozymes” are adapted to perform the same enzymatic functions as their non-extreme counterparts, but with greater versatility and adaptability to harsh conditions. These enzymes are found in extremophiles, organisms that thrive in extreme physical and/or chemical conditions. Extremophiles are found in some of the most severe environments on Earth, including hydrothermal vents, hypersaline lakes and pools, alkaline soda lakes, dry deserts, cold oceans, and volcanic areas.

Many industries are making efforts to move away from the use of harmful chemical processes. As a result, there is increasing biotechnological demand for enzymes that are stable and active under extreme conditions, such as those found in industrial processing. Over the past decade, screening, isolating, and optimizing the production of extremozymes has become one of the major areas of biotechnology research. The development of advanced, high-throughput screening and molecular biology techniques has facilitated the production of enzymes with extreme and optimized features. Despite these advances, however, the current enzyme toolbox is unable to meet industrial demands for processes requiring extreme conditions, such as high temperature, alkaline pH, low water activity, and resistance to metal ions. This Research Topic features some the latest progress in extremozyme research, with a focus on stability under extreme conditions and applicability to industrial processes.

Beginning with the first use of an enzymatic preparation for a commercial application, Sarmiento et al. review the history of enzyme discovery and the development of the market need for robust extremozymes. The authors then focus on the characteristics of psychrophilic, thermophilic, and hyperthermophilic enzymes and discuss their current applications in research and industrial areas, the challenges of working with extremozymes, and highlight future trends in the discovery and production of extremozymes.

Extremophilic enzymes play key roles in catalyzing commercially valuable chemical conversions without the production of toxic waste, which is the hallmark of traditional industrial chemical processes. These enzymes can be cloned and over-expressed, allowing for production of sufficient quantities for detailed biochemical and structural characterization and large-scale production for industrial processes. Littlechild describes several extremozymes with applications in biocatalysis, which have been developed for industrial processes, such as γ-lactamase, l-aminoacylase, and carboxypeptidase.

Among the different classes of extremozymes, thermophilic and thermostable enzymes are the most extensively sought after for industrial processes owing to their stability and activity at high temperatures. Thermophilic enzymes are also more resistant than their mesophilic counterparts to organic solvents, metal cations, and chemical denaturants. Artz et al. describe the biochemical and structural characterization of a thermostable mercuric ion reductase that remains active after prolonged incubation at temperatures greater than 90°C and potentially has a unique method of coordinating mercury ions. Bhalla et al. describe the purification and characterization of an extremely thermophilic xylanase with a half-life of 12 days at 70°C that exhibits activity on untreated lignocellulose, giving it great potential for application in biofuels production. Schröder et al. describe two unique, highly thermostable glycosyl hydrolases with β-glucosidase activity. Dennett and Blamey describe the production and purification of a thermophilic nitrilase that retains activity after prolonged incubation at 85°C. Finally, Zadvornyy et al. present the characterization of a thermophilic enolase from the green, non-sulfur bacterium *Chloroflexus aurantiacus* with maximal activity at 80°C and exhibiting structural properties that indicate it is thermally adapted.

Advances in metagenomics have contributed greatly to the production of novel extremozymes since these techniques can help overcome the limitations of culture-based approaches. Following the screening of a large-insert mixed genomic DNA library of pooled isolates of hyperthermophilic archaea from deep sea vents, Leis et al. isolated an extremely thermophilic endo-1,4-β-glucanase that showed maximal activity at 92°C and was active toward various substrates.

Alkaliphilic enzymes have increased resistance to alkali and other denaturing chemicals. The majority of alkaliphilic enzymes characterized to date are produced by extremely alkaliphilic *Bacillus* strains. Preiss et al. reviewed alkaliphilic bacteria and the bioenergetics aspects of survival in a dearth of protons. The review focuses on the bioenergetic challenges faced by alkaliphiles that carry-out proton-coupled oxidative phosphorylation and provides a detailed discussion of adaptations of alkaliphile F_1_F_o_-ATP synthases that support function at a low proton motive force.

The works highlighted in this Research Topic illustrate the continuing advancements in the field of extremozymes research, as well as the applicability of these enzymes to several industries. However, the potential of extremozymes is far from being fully understood and continued research efforts are still needed. The development of novel culture methods, suitable molecular tools, more efficient production processes, and improvements in novel technologies, such as functional metagenomic screenings, genome mining, and protein engineering, are key factors to fully unlocking the power of extremozymes. Advancing in this direction will greatly improve the yield and variety of extremozymes produced and their potential industrial applications.

## Author Contributions

Both authors have read and revised the manuscript.

## Conflict of Interest Statement

The authors declare that the research was conducted in the absence of any commercial or financial relationships that could be construed as a potential conflict of interest.
